# Undrained stability of dual tunnels in layered soils with different strength

**DOI:** 10.1038/s41598-022-14998-x

**Published:** 2022-06-24

**Authors:** Yongge Zeng, Tianqin Zeng, Gaoqiao Wu

**Affiliations:** 1 Shaoyang University, Shaoyang, 422000 People’s Republic of China; 2 Tsinghua University, Beijing, 100089 People’s Republic of China

**Keywords:** Engineering, Civil engineering

## Abstract

The stability of dual circle tunnels buried in layered soils with different shear strengths was investigated by using finite element limit analysis (FELA). The emphasis of this study is in quantitating that the existing tunnels affects the newly-built one, and design suggestions have been provided especially in the optimum location of construction. By imposing a FELA modelling, the variation trends of undrained bearing capacity with different influential factors, including the horizontal distance, vertical distance, the thickness of the top layer, the shear strength ratio of the layered soil, were further investigated. It is concluded that there exists an inclination-fixed worst-band, in which there would be a worst undrained stability once the bottom tunnel was constructed in the band. It is interested that the inclination seems constant by varying several factors but the horizontal distance would be changed with the soil properties. In addition, three patterns of collapse were summarized.

## Introduction

Tunnelings are frequently encountered in engineering practice due to the rapid urbanization, and the essential problem in tunneling engineering is the stability of both the construction itself and the stratum^1–15^. The majority of published research focuses on the single tunnel and parallel twin tunnel. Due to limited space and rising traffic demands, cases of a newly-built tunnel undercrossing an existing tunnel (e.g., the subway underpass) are indeed unavoidable. Under these conditions, the fine equilibrium of geostatic stresses would be disrupted, with the disturbance of pre-consolidation having a major detrimental effect on the stability of the existing tunnel. To the best of the author’s knowledge, there are only few literature as concerns this issue. Kiyosumi et al.^16^ employed commercial finite element software Plaxis to investigated the ultimate bearing capacity of strip footing lying on multiple voids. Four types of classic dual void configurations including symmetrical configuration, parallel configuration, serial configuration and offset configuration were analyzed in detail. The results indicated that for footing-dual void system, the void near the footing exerts much more influence than the other void on the footing stability. And the effect of voids will vanish once the footing-void distance beyond a critical distance. Xiao et al.^2^ developed adaptive finite element limit analysis (AFELA) code to investigate the influence of vertical relative distance between the double circular tunnels on the stability, soon afterwards the issue had been extended to the cases of square tunnels in rock mass^17^. In these cases, the assumption of uniform soils or rocks was made for simplification. However, the soil would be layered through long-term sedimentation processes in reality. It is worth noting that the failure patterns and stability of tunnels in two-layered soils is much different from that in single-layered soils. And a newly-built tunnel is inevitably constructed in different soil layer from the existing tunnel. In order to further guarantee the safety in construction, this study employed FELA to explore the stability of dual tunnels at different depth, special attention being focused on the effect of layered soils. By imposing the FELA modelling, the effects of various influential factors on the stability of tunnels were investigated, and an inclination-fixed worst-band was revealed to guide the site selection of newly-built tunnel near existing tunnel. Visualized shear dissipation nephograms were presented to reveal how the failure pattern will evolve with varying influential factors. And three typical failure patterns corresponding to different stability levels were summarized for deeper insight into the failure mechanism of the dual-tunnel system.

## Problem definition

**Figure 1:**
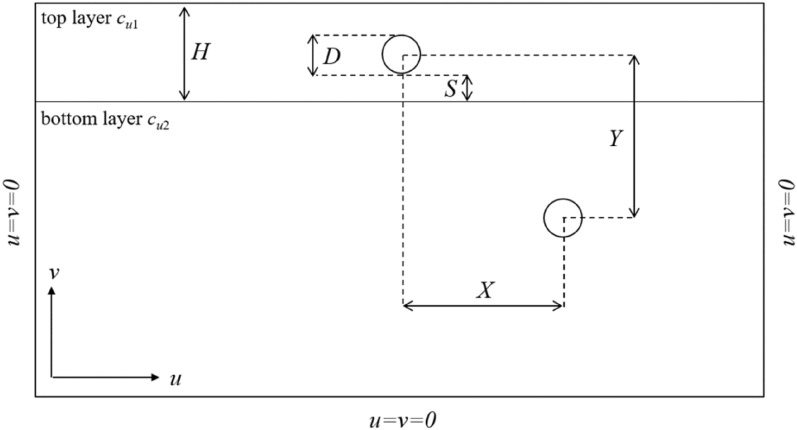
Problem definition.

Two circular tunnels with diameter *D* located in two-layers soil are defined, as sketched in Fig. 1. The dimensionless vertical and horizontal distance between tunnels is *Y*/*D* and *X*/*D* respectively. In the subsequent investigation, cases of *Y*/*D* = 2, 3, 4, 5, 6 and *X*/*D* = 0, 1, 2, 3, 4, 5, 6, 7 are considered. The dimensionless thickness of top layer is *H*/*D*, and the cases of *H*/*D* = 2 and 3 are analyzed in this study. And the distance between the top tunnel and the bottom layer is *S*/*D* which is fixed as 0.5 in this study to simplify calculations. For homogeneous soil, the bearing capacity can be expressed as a stability factor *N*_*c*_ = *σ*_*s*_/*c*_*u*_^13^. Hence, the stability factor *N*_*c*_ can be defined as follows

1 $$N_{c} = \frac{{\sigma_{s} }}{{c_{u1} }} = f\left( {\frac{H}{D},\frac{X}{D},\frac{Y}{D},\frac{\gamma D}{{c_{u1} }},\frac{{c_{u1} }}{{c_{u2} }}} \right)$$

where *σ*_*s*_ is the surcharge load, *c*_*u*1_, *c*_*u*2_ and *γ* are the undrained shear strength of top layer, bottom layer and unit weight of soil, which follows the Tresca failure criterion^3,11–13,18,19^. The unit weight is fixed as 20 kN/m^3^. And cases of *c*_*u*1_/*c*_*u*2_ = 0.25, 0.5, 0.75, 1, 1.5, 2, 3, 4, 5 are investigated in this study.

## FELA modelling

**Figure 2:**
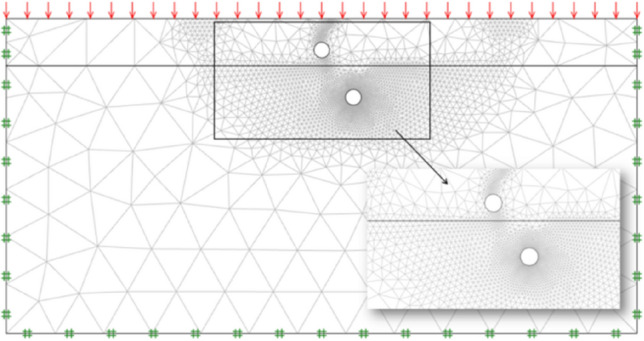
General view of numerical model.

As an accurate and efficient method, FELA was used to address geotechnical stability issues^1–3,7,9–22^. FELA method can acquire accurate ultimate loads by a combination of the limit theorems of plasticity and finite elements^23^. Strictly close predictions of lower bound (LB) and upper bound (UB) results can be obtained by reasonable construction of the statically admissible stress field and kinematically admissible velocity field, respectively. In view of the superiority of FELA method, a computational software Optum G2^24^ is employed in this study to investigate the ultimate bearing capacity and failure mechanisms of dual tunnels in layered soil with different strength. This program can refine precise mesh on the strength of the adaptive meshing technique. In this study, the number of initial mesh and finial mesh is set as 1000 and 5000 to obtain the accurate results and reasonable operation time, and the iteration of mesh refinement is set as 3 steps. The lateral and bottom boundaries are fixed in all directions to follow previous literature^1,2^. The size of soil field was set as 40B*20*B* which is large enough to avoid the boundary effect. And the general view of this FELA model is depicted in Fig. 2.

**Figure 3:**
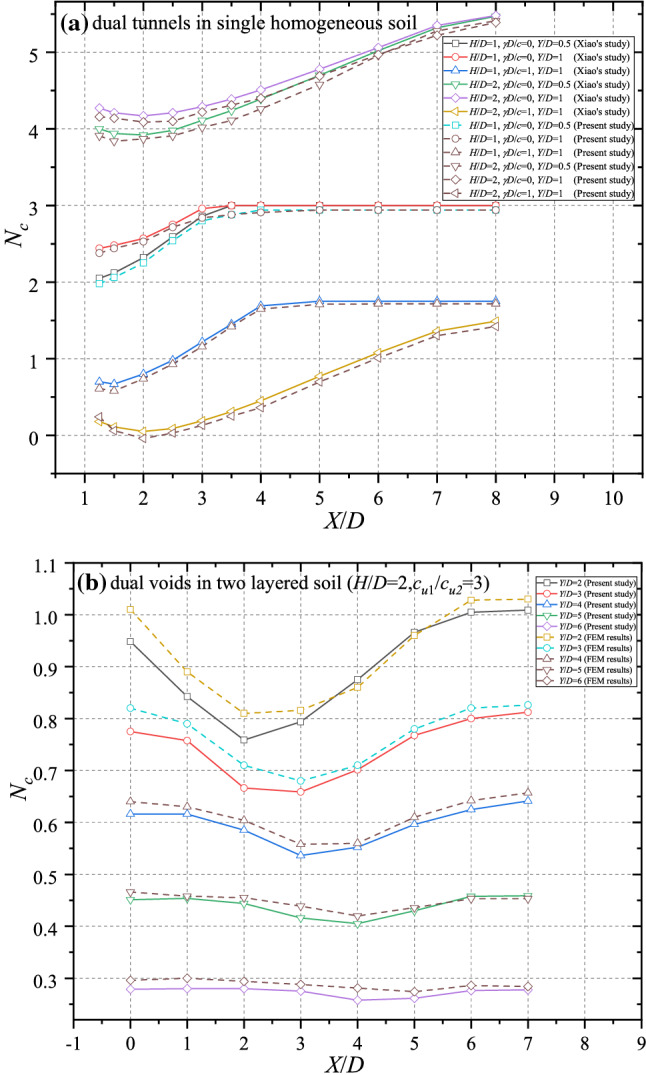
Comparison of *N*_*c*_ − *X*/*D* curves for (**a**) dual tunnels in homogeneous soil; (**b**) dual tunnels in two layered soil.

To ensure the reliability of the FELA modelling, the average value of UB and LB FELA results are compared with previous literature^2^ and FEM method. Figure 3a shows the cases of dual voids in single layer soil at different depth. It can be seen that the results of FELA agree well with Xiao’s results. Figure 3b shows the comparison between FELA results and FEM results obtained from FEM program Plaxis^2D^^25^. The FEM results covers all the cases by using FELA modelling, dual tunnels located in two layers of soil. The results obtained from FELA are similar to those from FEM for most cases. The maximum error between the two methods occurs in the case of *X*/*D* = 2, *Y*/*D* = 2. The maximum error, 5.7%, can be calculated by

2 $${\text{Error = }}\left| {\frac{{2\left( {\text{A - B}} \right)}}{{{\text{A}} + {\text{B}}}}} \right| \cdot 100{\text{\% }}$$

It is well known that one of the superiorities for FELA method is the adaptive meshing technique, providing a more reasonable mesh to obtain more accurate results than the FEM method. There would be discrepancies caused by different meshing methods, and a relatively small discrepancy (less than 5.7%) proves the reliability of FELA modelling.

## Results and discussions

The effect of influential factors presented in Eq. (1) on *N*_*c*_ are investigated as follows.

### Effect of the shear strength ratio c_u1_/c_u2_

**Figure 4:**
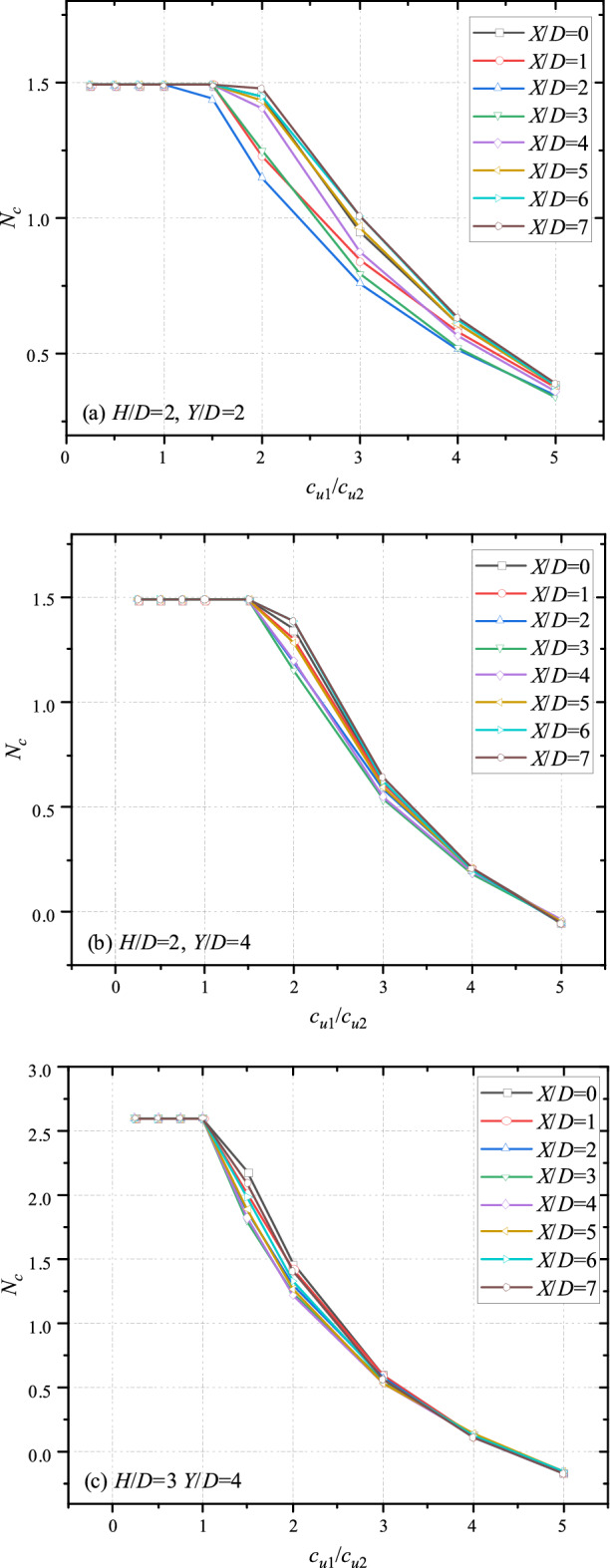
Variation of *N*_*c*_ with different *c*_u1_/*c*_u2_ and *X*/*D* for (**a**) *H*/*D* = 2, *Y*/*D* = 2; (**b**) *H*/*D* = 2, *Y*/*D* = 4 and (**c**) *H*/*D* = 3, *Y*/*D* = 4.

**Figure 5:**
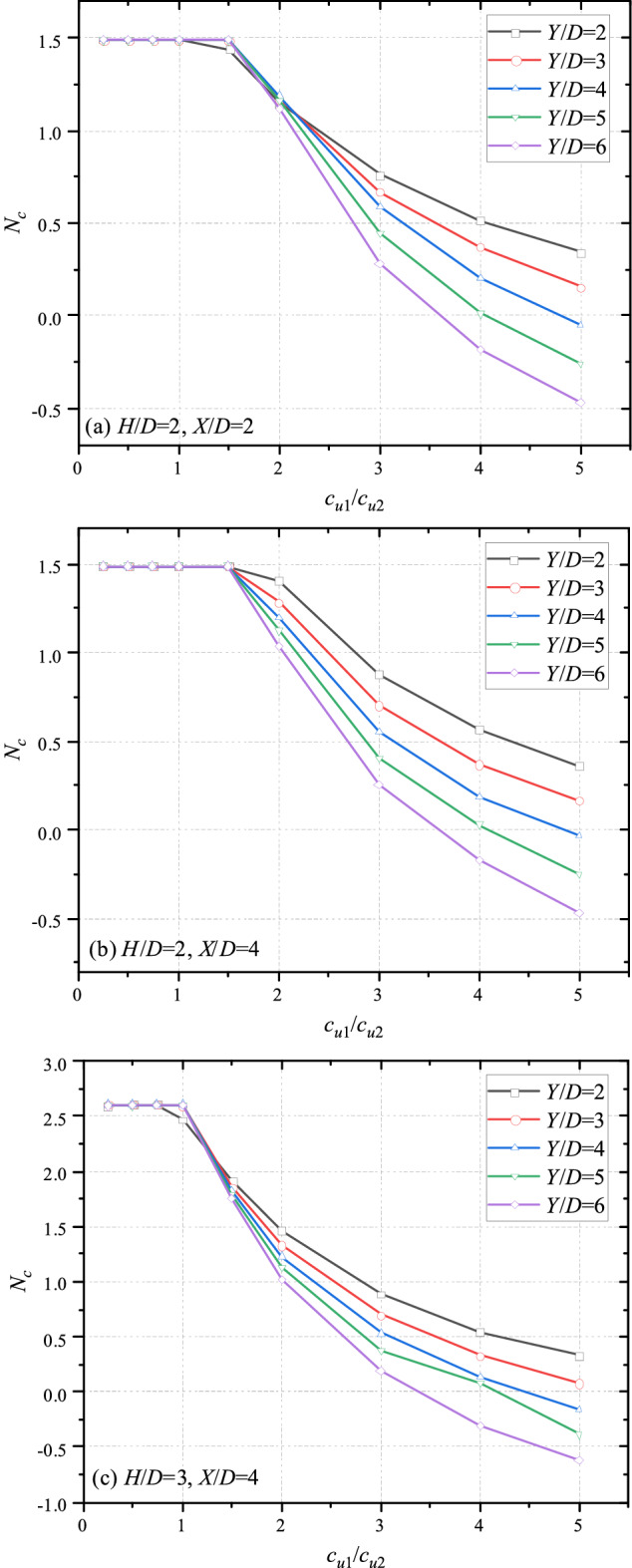
Variation of *N*_*c*_ with different *c*_u1_/*c*_u2_ and *Y*/*D* for (**a**) *H*/*D* = 2, *X*/*D* = 2; (**b**) *H*/*D* = 2, *X*/*D* = 4 and (**c**) *H*/*D* = 3, *X*/*D* = 4.

Figures 4 and 5 show the variation trends of undrained bearing capacity with different *c*_u1_/*c*_u2_ and horizontal/vertical distance between the double tunnels. It is obvious that for all cases, the bearing capacity keeps constant before *c*_u1_/*c*_u2_ = 1, indicating that the deeper tunnel has no effect on the bearing capacity. It reasons that the bottom layer is stiff enough to resist the distributed load which can lead the collapse of the shallower tunnel. When *c*_u1_/*c*_u2_ > 1, the bearing capacity decreases with the increase of *c*_u1_/*c*_u2_, indicating that the influence of soft bottom layer becomes significant. To sketch the trend accurately, a term, knee point, is introduced to define the point where the bottom layer starts to affect the system. It can be seen that the knee point is at greater shear strength ratio (*c*_u1_/*c*_u2_ = 2) with deeper depth of tunnel (*H*/*D* = 2, *Y*/*D* = 4), whereas the knee point is at *c*_u1_/*c*_u2_ = 1.5 for cases of *H*/*D* = 2, *Y*/*D* = 2. And for cases of *H*/*D* = 3 (depicted in Fig. 4c), the knee point is at *c*_u1_/*c*_u2_ = 1.5, which is smaller than the *c*_u1_/*c*_u2_ of knee point for cases of *H*/*D* = 2 (depicted in Fig. 4b). Furthermore, it can be seen that the bearing capacity in Fig. 4 decrease with the increase of *c*_u1_/*c*_u2_, and all curves tend to overlap with the increase of *c*_u1_/*c*_u2_. It indicates that the effect of *X*/*D* becomes insignificant if the top layer is much stiffer than the bottom layer. And the overlap phenomenon is more dominant for cases of greater *H*/*D* and *Y*/*D*.

It can be observed from Fig. 5a,b that the knee point is at *c*_u1_/*c*_u2_ = 1.5 for cases of *X*/*D* = 2 while the knee point of *X*/*D* = 4 is at *c*_u1_/*c*_u2_ = 2. The knee point of *H*/*D* = 3 (depicted in Fig. 5c) occurs at a less value of *c*_u1_/*c*_u2_, compared to the case of *H*/*D* = 2 (depicted in Fig. 5b), similar to Fig. 4. Based on the variation trends of Figs. 4 and 5, it can be predicted that for cases of thicker top layer or relatively closer bottom tunnel, the knee point would appear at a less value of *c*_u1_/*c*_u2_. And the scatter of group of curves in Fig. 5 raises with the increase of *c*_u1_/*c*_u2_. It reasons that the deeper the tunnel is, the greater the soil load it bears. This trend becomes more significant with the increase of *c*_u1_/*c*_u2_. And the soil weight would lead to a negative value of bearing capacity. The negative value means that the tunnel requires extra lining to maintain its stability.

### Effect of the vertical distance of tunnels Y/D

**Figure 6:**
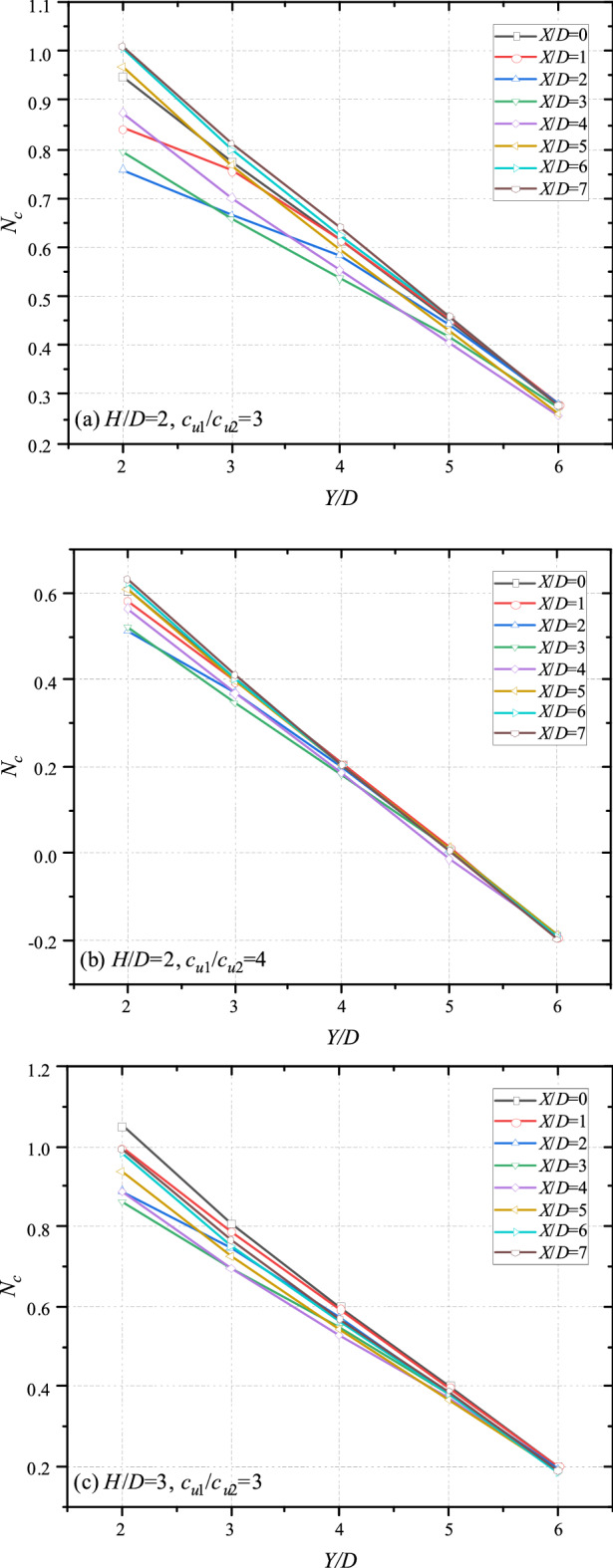
Variation of *N*_*c*_ with different *Y*/*D* and *X*/*D* for (**a**) *H*/*D* = 2, *c*_u1_/*c*_u2_ = 3; (**b**) *H*/*D* = 2, *c*_u1_/*c*_u2_ = 4 and (**c**) *H*/*D* = 3, *c*_u1_/*c*_u2_ = 3.

Figure 6 presents the variation trend of bearing capacity with different *Y*/*D*. It can be seen that for nearly all cases, the depth of bottom tunnel affects the bearing capacity linearly. As expected, all curves of Fig. 6 show a downtrend with the increase of *Y*/*D*, which is caused by the large unit weight of soil. And the curves of Fig. 6b are highly coincidence, it illustrates that the influence of *X*/*D* becomes weaker with greater *c*_u1_/*c*_u2_ and less *H*/*D*. Furthermore, it is interesting that there are some cross points in Fig. 6. It indicates that the bearing capacity would not increase with the increase of *X*/*D* monotonously. The detailed investigation of this phenomenon would be presented in the next section.

### Effect of the horizontal distance of tunnels X/D

**Figure 7:**
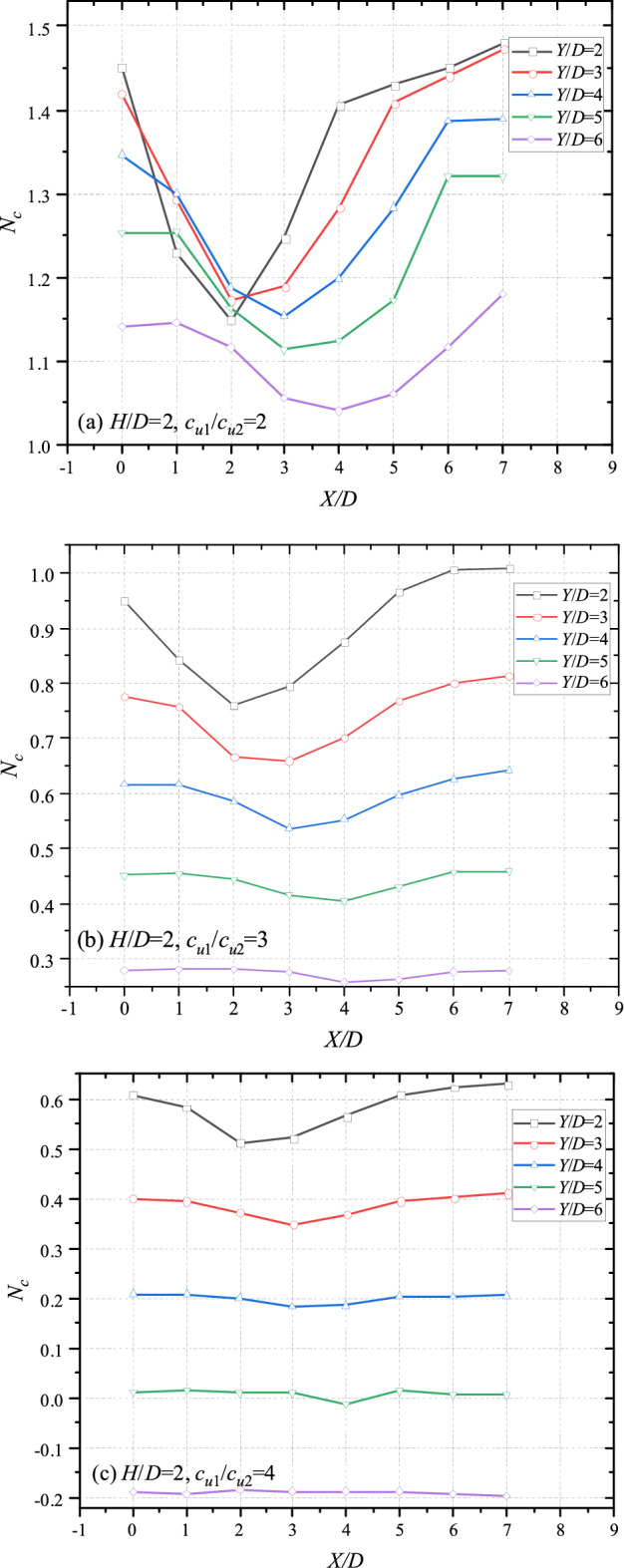
Variation of *N*_*c*_ with different *X*/*D* and *Y*/*D* for (**a**) *H*/*D* = 2, *c*_u1_/*c*_u2_ = 2; (**b**) *H*/*D* = 2, *c*_u1_/*c*_u2_ = 3 and (**c**) *H*/*D* = 2, *c*_u1_/*c*_u2_ = 4.

**Figure 8:**
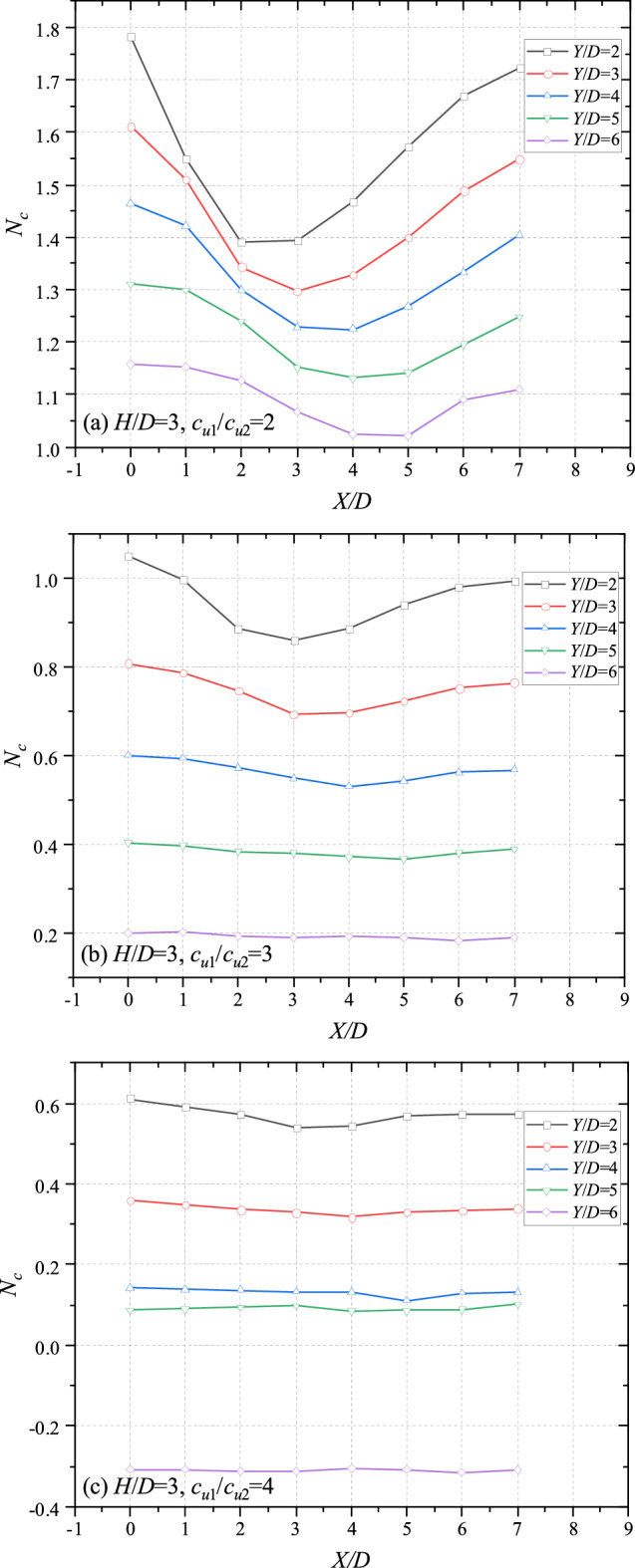
Variation of *N*_*c*_ with different *X*/*D* and *Y*/*D* for (**a**) *H*/*D* = 3, *c*_u1_/*c*_u2_ = 2; (**b**) *H*/*D* = 3, *c*_u1_/*c*_u2_ = 3 and (**c**) *H*/*D* = 3, *c*_u1_/*c*_u2_ = 4.

**Figure 9:**
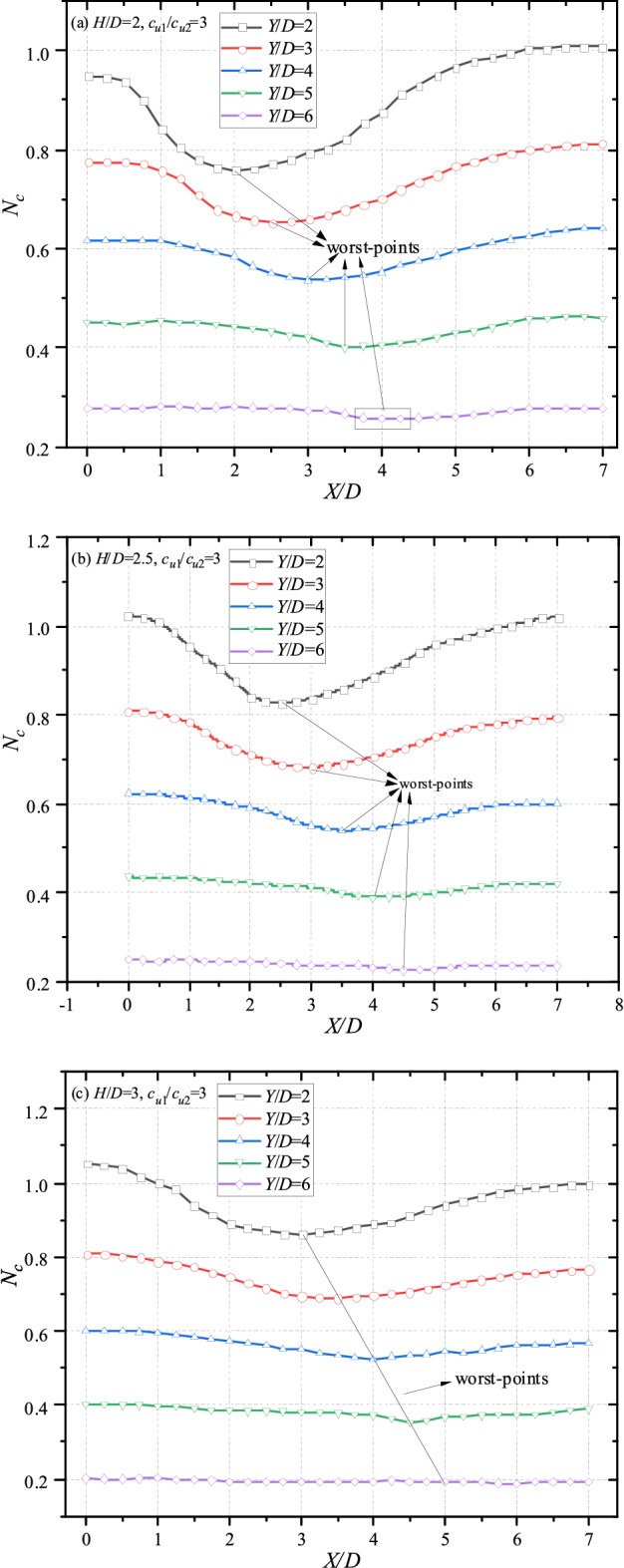

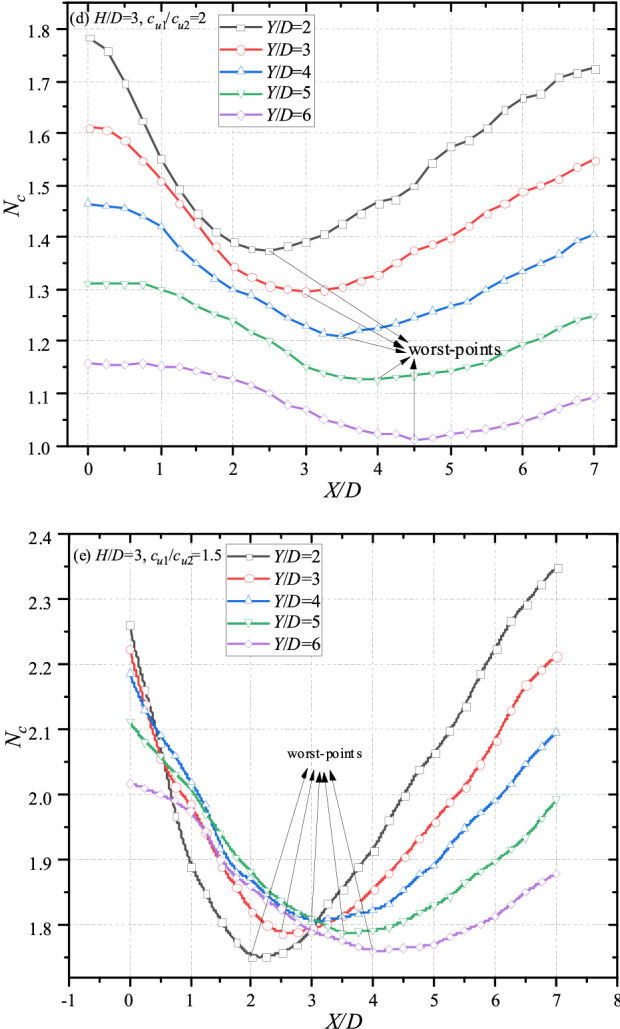
Variation of *N*_*c*_ with different *X*/*D* and *Y*/*D* for (**a**) *H*/*D* = 2, *c*_u1_/*c*_u2_ = 3; (**b**) *H*/*D* = 2.5, *c*_u1_/*c*_u2_ = 3; (**c**) *H*/*D* = 3, *c*_u1_/*c*_u2_ = 3; (**d**) *H*/*D* = 3, *c*_u1_/*c*_u2_ = 2 and (**e**) *H*/*D* = 3, *c*_u1_/*c*_u2_ = 1.5.

The effects of *X/D* on bearing capacity are shown in Figs. 7 (*H*/*D* = 2) and 8 (*H*/*D* = 3). Commonly, for cases of double tunnels/voids, previous studies indicate that the farther the distance between voids is, the greater the bearing capacity becomes. However, for cases of layered soils, majority of curves in Figs. 7 and 8 are concave. It means that the minimum of bearing capacity would not appears when *X*/*D* = 0. It can be observed that the valley of each figure would move farther towards the top tunnel with the increase of *Y*/*D*. For the cases of *H*/*D* = 2 and *c*_u1_/*c*_u2_ = 2 (depict in Fig. 7a), the minimum bearing capacity of *Y*/*D* = 2 and *Y*/*D* = 3 takes place at *X*/*D* = 2, whereas the minimum bearing capacity of *Y*/*D* = 4 and *Y*/*D* = 5 at *X*/*D* = 3. The lowest value of *Y*/*D* occurs at *X*/*D* = 4. This phenomenon also can be observed in other cases of Figs. 7 and 8. It is worth noting that the curves are likely to be horizontal with the increase of *c*_u1_/*c*_u2_, especially for cases of great *Y*/*D*. It reasons that the strength of bottom layer becomes weaker with the increasing *c*_u1_/*c*_u2_. And the influence of *X*/*D* would be vanished. According to the tendency illustrated in Figs.7 and 8, it can be deduced that there would be of a worst-band, in which the bearing capacity of the system would be the lowest if the bottom tunnel located. Figures 7 and 8 can be redrawn as Fig. 9 to obtain a deeper insight into the worst-band by scaling the x axial (varying from *X*/*D* = 1 per unit to *X*/*D* = 0.25 per unit).

### Detailed investigations of the worst-band

**Figure 10:**
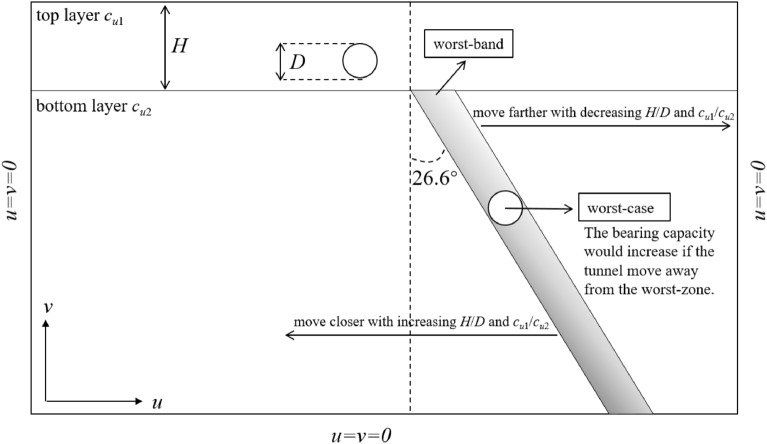
The sketch of the worst-band.

The re-scaling graphs are shown in Fig. 9 with different *H*/*D*, *c*_u1_/*c*_u2_ = 2. It can be seen from Fig. 9a that the worst-points with different *Y*/*D* locate at *X*/*D* = 2, 2.5, 3, 3.5, 4 respectively. And the *X*/*D* of worst-points is equidifferent. Namely, the worst-band of Fig. 9a could be a straight line. Interestingly, the worst-bands are also straight with the increasing thickness of top layer (*X*/*D* = 2.5, 3, 3.5, 4, 4.5 for cases of *H*/*D* = 2.5; *X*/*D* = 3, 3.5, 4, 4.5, 5 for cases of *H*/*D* = 3). That is to say, for worst cases with adjacent *Y*/*D* (e.g. *Y*/*D* = 2 and 3, *Y*/*D* = 3 and 4), the gradient of equation ${{\Delta X} \mathord{\left/ {\vphantom {{\Delta X} {\Delta Y}}} \right. \kern-\nulldelimiterspace} {\Delta Y}}$ would keep constant. In other words, the angle between the worst-line and the vertical line keeps constant, being roughly 26.6°(arctan0.5). On account of this, an angle-fixed worst-band (depicted in Fig. 10) can be defined, which would move farther away from the top tunnel with the thicker and stiffer top layer. The bearing capacity of whole system would be the worse if the bottom tunnel locates near to the worst-band. The worst cases in Fig. 9a–e are presented in Table 1 for a better understanding, obviously, the intervals between adjacent curve are all equal to 0.5, further revealing the existence of the worst-band.

**Table1:** *X*/*D* of worst cases with different parameters.

*Y*/*D*	*X*/*D*				
	*H*/*D* = 2, *c*_u1_/*c*_u2_ = 3	*H*/*D* = 2.5, *c*_u1_/*c*_u2_ = 3	*H*/*D* = 3, *c*_u1_/*c*_u2_ = 3	*H*/*D* = 3, *c*_u1_/*c*_u2_ = 2	*H*/*D* = 3, *c*_u1_/*c*_u2_ = 1.5
2	2	2.5	3	2.5	2
3	2.5	3	3.5	3	2.5
4	3	3.5	4	3.5	3
5	3.5	4	4.5	4	3.5
6	4	4.5	5	4.5	4
Interval	0.5	0.5	0.5	0.5	0.5

Based on these observations, helpful suggestions can be drawn that if there would be a newly-built tunnel near an existing tunnel, the site selection of the newly-built tunnel should locate away from the worst-band to guarantee an optimum bearing capacity.

In addition, it can be observed from Figs. 7, 8 and 9 that the curves show a downtrend with the increase of *X*/*D*, and the bearing capacity of *X*/*D* = 0 is the greatest. Generally, the closer the distance between dual tunnels/voids is, the greater the interaction between them becomes. And the interaction between dual tunnels can weaken the stability of the system, which is inconsistent with the common sense. In view of this inconsistency, analyses of failure mechanisms would be presented in next section to explain this phenomenon.

## Failure mechanism

For deeper insight into the bearing capacity of whole system, several typical failure patterns are discussed in this section.

**Figure 11:**
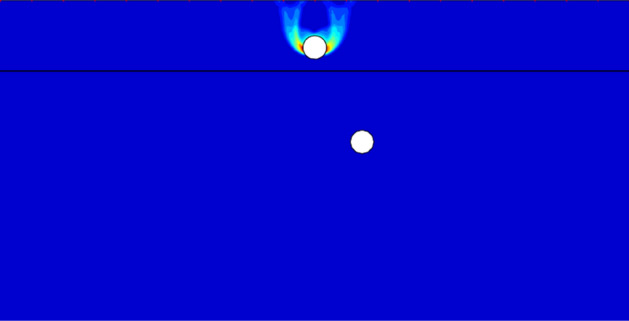
The UB failure mechanism of *c*_u1_/*c*_u2_ = 0.5, *X*/*D* = 2, *Y*/*D* = 4.

Figure 11 shows the failure mechanism with *c*_u1_/*c*_u2_ = 0.5, *X*/*D* = 2, *Y*/*D* = 4. It is obvious that the failure zone is confined into the top layer, and the failure pattern is the typical side wall failure of single tunnel. The reason for this failure pattern is when *c*_u1_/*c*_u2_ < 1, the bottom layer is stiff enough to avoid the collapse before the top tunnel is totally collapsed. That is to say, the failure pattern and bearing capacity would keep invariable if the *c*_u1_/*c*_u2_ < 1, which corresponds to tendencies of Figs. 4 and 5.

**Figure 12:**
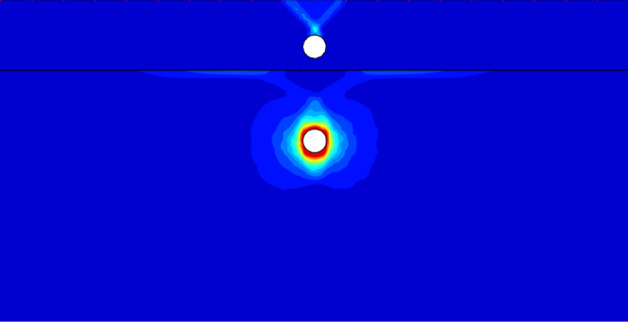
The UB failure mechanism of *c*_u1_/*c*_u2_ = 3, *X*/*D* = 0, *Y*/*D* = 4.

Figure 12 shows a special failure pattern with *c*_u1_/*c*_u2_ = 3, *X*/*D* = 0, *Y*/*D* = 4. It can be seen that there exists similar triangular wedge above each tunnel. But the reasons for these two triangular wedge are different. For the top wedge, it is a typical failure pattern of a single void under surcharge loading, which means that the top of the tunnel is under pressure stress. And the top compression collapse is the primary cause of the top tunnel failure. As to the occurrence of bottom wedge, it reasons that the tunnel in the top stiffer soil bear the surcharge loading and prevent a deeper load transmission to the bottom layer. Hence, it can be observed that there has no failure curve between these two tunnels, indicating that the collapse of top tunnel is depended upon the surcharge loading but not relies on the bottom tunnel. In addition, it can be seen that the deformation around the bottom tunnel is distinct. Namely, the circumferential squeezing to the bottom tunnel is the dominating collapse of this system.

**Figure 13:**
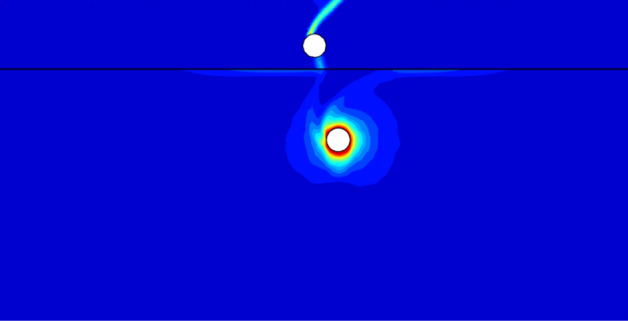
The UB failure mechanism of *c*_u1_/*c*_u2_ = 3, *X*/*D* = 1, *Y*/*D* = 4.

Figure 13 presents the failure mechanism with *c*_u1_/*c*_u2_ = 3, *X*/*D* = 1, *Y*/*D* = 4. It is obvious that the primary collapse is also the circumferential squeezing to the bottom tunnel. However, with the shift of the bottom tunnel, it can be observed that there appears a light failure curve connecting the top and bottom tunnel. It indicates that the softer bottom tunnel has a little influence on the top tunnel, which would induce the decrease of bearing capacity. Furthermore, it can be seen there only has single failure curve between the ground and the top tunnel. It means that the shift of the bottom tunnel can lead to an asymmetric deformation of the top tunnel.

**Figure 14:**
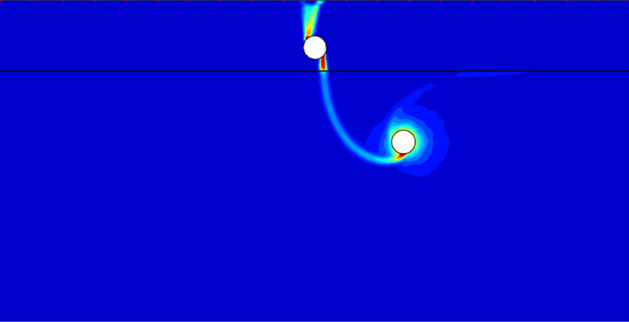
The UB failure mechanism of *c*_u1_/*c*_u2_ = 3, *X*/*D* = 4, *Y*/*D* = 4.

Figure 14 shows the worst case of *Y*/*D* = 4. It can be found that there are two features which cause the worst stability of whole system: (1) an obvious failure curve between the dual tunnels, which means the bottom tunnel has a considerable negative effect on the top tunnel; (2) an obvious failure curve between the top tunnel and the ground surface, it means that the surcharge loading also has great influence on the top tunnel. And the combination of strong interaction between the tunnels and the great influence of surcharge loading lead to a large deformation of the top tunnel, which would cause the worst stability.

**Figure 15:**
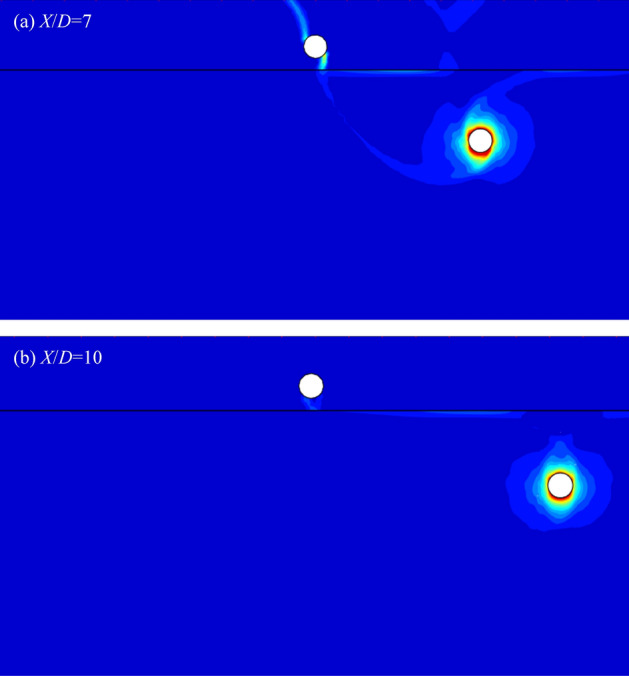
The UB failure mechanism of *c*_u1_/*c*_u2_ = 3, *Y*/*D* = 4 for (**a**) *X*/*D* = 7 and (**b**) *X*/*D* = 10.

Figure 15 shows the failure mechanisms with *c*_u1_/*c*_u2_ = 3, *Y*/*D* = 4, *X*/*D* = 7 and 10. It can be found from Figs. 14 and 15a that the interaction between the tunnels becomes weaker with the increase of *X*/*D*. The failure curve between the top tunnel and the ground surface also lighter than the worst case (depicted in Fig. 14). And the circumferential squeezing to the bottom tunnel becomes to the primary collapse again. With the farther shift of bottom tunnel (*X*/*D* = 10), it can be seen from Fig. 15b that there is no failure curve between the ground and the top tunnel, which illustrates that the surcharge loading has no influence on the top tunnel. And all the deformation of top tunnel is caused by the collapse of bottom tunnel, and the interaction between the dual tunnels is very weak. In view of this, it can be predicted that the circumferential squeezing to the bottom tunnel would be the only collapse type if the horizontal distance between the tunnels is far enough.

It is worth noting that there are three typical failure patterns can be summarized to describe the interaction between the dual tunnels. Figure 12 depicts a situation that there has no interaction between the tunnels for cases of a much close horizontal distance between the dual tunnels, which is defined as ‘independent double tunnel failure’. And Figs. 13 and 14 show series familiar failure patterns, which defined as ‘combined tunnel failure’, corresponding to interactions of different strength. Figure 15 shows a failure pattern that the interaction between tunnels almost disappear with the increase of horizontal distance between tunnels, which defined as ‘primary bottom tunnel failure’. And the variation of failure mechanisms presented in Figs. 12, 13, 14 and 15 can further verify the variation trend (downtrend firstly, then uptrend) in Figs. 7, 8 and 9. Furthermore, these failure patterns are representative which include all the failure patterns of this study. Limited by the length of paper, the failure mechanisms with more *H*/*D*, *Y*/*D*, *X*/*D* and *c*_u1_/*c*_u2_ wouldn’t discuss there.

## Conclusions

This study employs FELA to investigate the undrained stability of dual tunnels locate in layered soils. Several influential factors have been investigated, including the thickness of top layer (*H*/*D*), the horizontal and vertical distance between the dual tunnels (*X*/*D* and *Y*/*D*) and the shear strength ratio (*c*_u1_/*c*_u2_). Representative failure patterns are also discussed for deeper insight into the bearing capacity of whole system. Based on these results from FELA, some conclusions can be drawn as follows (it should be noted that the following conclusions are all based on *c*_u1_/*c*_u2_ ≥ 1, because the bearing capacity and the failure mechanism are invariable for cases of *c*_u1_/*c*_u2_ < 1):

1. The curve of stability shows a downtrend initially than uptrend with the increase of horizontal distance between the dual tunnels;
2. There is an angle-fixed worst-band; the closer the distance between the bottom tunnel and the worst-band is, the worse the stability of the whole system becomes;
3. The worst-band would move farther away from the top tunnel with the increase of *H*/*D* and *c*_u1_/*c*_u2_;
4. Three typical failure patterns are summarized to reveal the interaction between the two tunnels. The patterns can further verify the variation of bearing capacity.

## Data Availability

The datasets used and/or analysed during the current study available from the corresponding author on reasonable request.
